# Cooling-Rate Computer Simulations for the Description of Crystallization of Organic Phase-Change Materials

**DOI:** 10.3390/ijms232314576

**Published:** 2022-11-23

**Authors:** Victor M. Nazarychev, Artyom D. Glova, Sergey V. Larin, Alexey V. Lyulin, Sergey V. Lyulin, Andrey A. Gurtovenko

**Affiliations:** 1 Institute of Macromolecular Compounds, Russian Academy of Sciences, Bolshoi Prospect V.O. 31, 199004 St. Petersburg, Russia; 2 Soft Matter and Biological Physics Group, Technische Universiteit Eindhoven, P.O. Box 513, 5600 MB Eindhoven, The Netherlands

**Keywords:** organic phase-change materials, paraffins, computer modeling, molecular dynamics, cooling rate, crystallization enthalpy, thermal conductivity

## Abstract

A molecular-level insight into phase transformations is in great demand for many molecular systems. It can be gained through computer simulations in which cooling is applied to a system at a constant rate. However, the impact of the cooling rate on the crystallization process is largely unknown. To this end, here we performed atomic-scale molecular dynamics simulations of organic phase-change materials (paraffins), in which the cooling rate was varied over four orders of magnitude. Our computational results clearly show that a certain threshold (1.2 × 10^11^ K/min) in the values of cooling rates exists. When cooling is slower than the threshold, the simulations qualitatively reproduce an experimentally observed abrupt change in the temperature dependence of the density, enthalpy, and thermal conductivity of paraffins upon crystallization. Beyond this threshold, when cooling is too fast, the paraffin’s properties in simulations start to deviate considerably from experimental data: the faster the cooling, the larger part of the system is trapped in the supercooled liquid state. Thus, a proper choice of a cooling rate is of tremendous importance in computer simulations of organic phase-change materials, which are of great promise for use in domestic heat storage devices.

## 1. Introduction

An accurate description of phase transformations is critical for molecular systems in which the phase transition is a key process from the point of view of their practical applications. The well-known representatives of such systems are organic phase-change materials (PCM). Typical organic PCMs, such as paraffins, are characterized by large latent heat of fusion, chemical stability, and relatively low cost [1,2]. These materials can adsorb and release a large amount of heat upon phase transitions (crystallization or melting) and are of great promise for use in domestic heat storage devices. Such devices are essential for the rational use of thermal energy, which has become an increasingly important issue in view of environmental protection, energy conservation, and reduction of carbon emissions [3]. It is, therefore, crucial to describe the phase transition itself and related changes in the thermophysical properties of PCMs as accurately as possible. This allows one to assess the thermal performance of PCM-based heat storage devices and to design novel devices with improved characteristics.

In experiments to study the phase transition in the organic PCMs, the differential scanning calorimetry (DSC) technique is widely used. In DSC experiments, a sample is continuously cooled/heated, and the heat flow is measured as a function of temperature. At the crystallization/melting point, the heat flow curve demonstrates a sharp maximum/minimum. As for paraffin samples, it was repeatedly shown that cooling/heating rate could affect their thermophysical and structural properties [4,5,6,7,8]. In particular, Hammami et al. reported an increase in the crystallization temperature for a series of seven n-alkanes upon decreasing the cooling rate from 10 to 1 K/min [5,6]. Lazaro et al. showed that for an n-octadecane sample, the heating rate should be lower than 0.5 K/min to eliminate the effects of the heating rate on the results [8]. Louanate et al. studied the nonisothermal crystallization behavior of technical grade paraffin wax and showed that the peaks of the crystallization curves became broader and shifted to lower temperatures when the cooling rate was increased from 0.3 to 20 K/min [4]. Lastly, Abdi et al. focused on the thermophysical properties of n-octadecane and n-eicosane and varied the cooling/heating rates from 0.025 to 0.5 K/min [7]. In the case of n-eicosane, the authors found that slowing down the cooling led to an increase in the crystallization enthalpy as well as to a shift of peaks of specific heat curves to higher temperatures. All in all, DSC experiments clearly demonstrated that cooling/heating rates impact the crystallization/melting behavior of paraffins, and care has to be taken to obtain accurate and reproducible results.

When it comes to computer modeling, the situation becomes even more involved. To study the nonisothermal crystallization of a paraffin sample, cooling-rate computer simulations are normally employed. In these simulations, the temperature of a sample is decreased in a stepwise manner; the crystallization temperature is defined at the point when an abrupt change in a certain physical characteristic occurs (e.g., mass density). Here, one can think of at least two issues. The first one is related to the time scales accessible for computer modeling. In general, atomistic computer simulations are of particular interest as they provide unprecedented insight into the structure and properties of molecular systems. At the same time, such high-resolution simulations are computationally demanding and often limited to microsecond time scales. This implies that the cooling rate in atomistic simulations could be 10^9^ K/min at best, which is nine orders of magnitude larger than that in experiments. In practice, one should always keep this difference in mind when comparing simulation results with experimental data. For instance, fast cooling of a paraffin sample in simulations could lead to supercooling when a part of the system does not crystallize at temperatures lower than the crystallization point. Such supercooling is not typical for paraffins, questioning, therefore, the reliability of computer simulations. Note, however, that supercooling constitutes a major problem for domestic heat storage devices where PCM emulsions (rather than pure materials) are used [9], so the cooling/heating rates could also matter for heat storage. The second issue is similar to what was discussed above for DSC experiments and related to the impact of the cooling rate on the computational results. Given the well-documented effects of the cooling rate in experiments, it is rather surprising that—to the best of our knowledge—there have been no published simulation studies on the influence of the cooling rate on the crystallization and related physical properties of paraffins.

Indeed, most cooling-rate computer simulations of paraffins (n-alkanes) employed cooling with a single rate, often without any explanations behind their choice of the rate [10,11,12,13]. There is a limited number of relevant studies on the crystallization of polyethylene which has the same chemical structure as n-alkanes but much longer chain lengths. Yamamoto et al. showed that rapid cooling of a polyethylene melt led to poorly crystallized and even nearly amorphous samples [14]. In addition, it was demonstrated that decreasing the cooling rate improves the crystallinity of a polyethylene sample. Ramos et al. performed cooling-rate atomistic simulations to study the crystallization of polyethylene; the authors varied the cooling rates in the range from 3 × 10^9^ to 6 × 10^10^ K/min [15]. It was shown that the peak of the specific heat capacity is shifted toward lower temperatures, and the transition becomes broader when the cooling rate is increased. Furthermore, the authors reported a decrease in the degree of crystallinity by speeding up the sample cooling [15]. Overall, the cooling rate was shown to significantly affect the crystallization process in cooling-rate computer simulations of polyethylene. Given the similarity in the chemical structure of polyethylene and paraffins, corresponding systematic studies of cooling-rate-related effects are much needed for paraffins too. Surprisingly, there is a complete lack of such studies despite the importance of paraffins as promising materials for heat storage devices. To this end, in our paper, we report the first computational study in which the impact of cooling rate is systematically explored. In particular, we demonstrate for the first time that too fast cooling could lead to a completely inaccurate description of the crystallization process in computer simulations.

In this paper, we employ cooling-rate computer simulations to characterize the crystallization of paraffins, an important representative of organic phase-change materials. In our simulations, the rate of cooling will be systematically varied, which allows us, for the first time, to unlock the impact of the cooling rate on the crystallization behavior and the properties of paraffins. For our purposes, we chose to consider n-eicosane (C_20_H_42_); this short n-alkane was shown to have great potential for use in domestic heat storage devices [1]. Recently, the properties of n-eicosane have been the subject of extensive computational studies with the use of 10 different atomistic models [16,17,18,19]. Furthermore, experimental data are also available for this n-alkane, which is very important in terms of validating simulation results. As we proceed to show, crystallization, as well as many thermophysical, structural, and dynamic properties of n-eicosane, depend critically on the rate with which cooling is performed in computer simulations. This implies that the cooling rate is one of the most important parameters of computer modeling of paraffins, along with force fields [16,17,18,19] and various algorithms for calculating thermal conductivity [18,19]. Our computational findings could therefore serve as a basis for an accurate description of the crystallization behavior of organic PCMs such as paraffins. In particular, they allow one to perform a comparative analysis of the results of earlier computational studies of paraffins, in which different cooling rates were considered.

## 2. Results and Discussion

### 2.1. Crystallization and Thermophysical Properties

**Figure 1:**
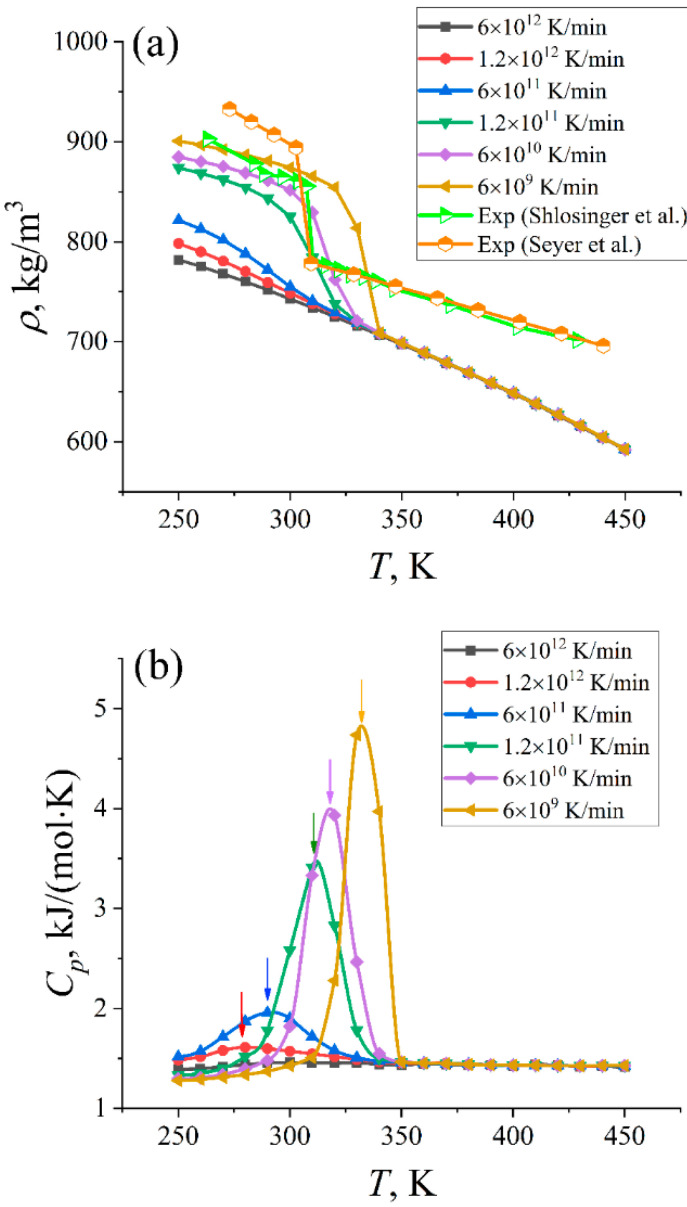
(**a**) Mass density *ρ* of n-eicosane samples as a function of temperature *T* for different cooling rates. Error bars (standard errors) are of the size of symbols and are not shown. For the sake of comparison, the experimental data [20,21] are also shown. (**b**) Molar specific heat capacity *C_p_* as a function of temperature for different cooling rates. The peaks corresponding to the crystallization of n-eicosane samples are marked by arrows.

Experimentally, crystallization, i.e., the phase transition from the liquid to a crystalline state, is characterized by an abrupt change in the temperature dependence of the sample’s physical properties. In particular, this is the case for the mass density of paraffin samples [20,21] (see Figure 1a). The point at which this abrupt change occurs is defined as the crystallization temperature. For n-eicosane, its value was found to be 310 K [1].

In our computer simulations, we cooled n-eicosane samples down from 450 to 250 K with different rates and measured the mass density *ρ* as a function of temperature *T*; the results are presented in Figure 1a. First off, it is seen that all *ρ(T)*-curves coincide in the high-temperature domain (*T* > 350 K). At lower temperatures, one can observe a rapid increase in the mass density (accompanied by a drop in the free volume). This significant change in the mass density is directly related to the crystallization of a paraffin sample. The overall picture turns out to be sensitive to the cooling rate: the transition is shifted to lower temperatures when the cooling rate increases, which is in line with experimental data [4,5,6,7,8]. The shape of the *ρ(T)*-curves also differ considerably in the low-temperature domain for the systems, depending on the cooling rate. In fact, all *ρ(T)*-curves in Figure 1a could be divided into two groups. The first one includes the three slowest cooling rates *γ_c_* (6 × 10^9^, 6 × 10^10^, and 1.2 × 10^11^ K/min) and could be called a “slow cooling group.” At these cooling rates, one can witness a relatively steep increase in the mass density when temperature drops, a behavior which closely resembles the picture observed in the experiment (see Figure 1a). In contrast, a “fast cooling group” with the three largest cooling rates (6 × 10^11^, 1.2 × 10^12^, and 6 × 10^12^ K/min) does not show a pronounced change in the *ρ(T)*-curves in the low-temperature domain. However, some bending of the curves for the cooling rates 6 × 10^11^ and 1.2 × 10^12^ K/min can still be distinguished, as illustrated in Figure S1, where we presented the results for paraffin’s cooling extended to *T* = 190 K (except for the system with the smallest cooling rate). Nevertheless, it is worth noticing that cooling the sample with the fastest rate of 6 × 10^12^ K/min completely suppresses paraffin crystallization.

Given the smoothness of *ρ*(*T*)-curves for the systems with high cooling rates, it is nontrivial to extract crystallization temperatures from the mass density profiles of samples. An alternative is to consider the specific heat capacity *C_p_* of a system [15]; the position of its peak can directly be related to the crystallization temperature. The results for the specific heat capacity of paraffin samples cooled down with different rates are shown in Figure 1b. Again, the *C_p_(T)*-curves could be divided into two groups, similar to *ρ(T)*-curves. A “slow cooling group” is characterized by the high and bell-shaped curve of the specific heat capacity as a function of temperature. In turn, *C_p_(T)*-curves of a “fast cooling group” are much lower and wider (see Figure 1b). From the position of the *C_p_(T)* peaks, we were able to extract crystallization temperatures for 5 systems cooled down at different rates (see Table 1). The only exception is a system with the highest cooling rate of 6 × 10^12^ K/min, which is another sign that a paraffin sample does not crystallize at such fast cooling. Overall, the crystallization temperature increases when the sample cooling slows down. This computational finding is in qualitative agreement with the experimental data available [4,5,6,7,8]. It is noteworthy that the effect of the cooling rate on the crystallization is opposite to the one observed for the glass transition of polymers, where the glass transition temperature drops when the cooling rate decreases [22]. In our case, the crystallization is connected to nucleation, which is, in turn, promoted by slower cooling so that the crystallization starts at higher temperatures. We also note that the crystallization temperature evaluated from simulations for a system with the smallest cooling rate exceeds that measured in the experiment (see Table 1). This feature is typical for all-atom force fields of paraffins, as they are known to overestimate the crystallization temperature [16].

**Figure 2:**
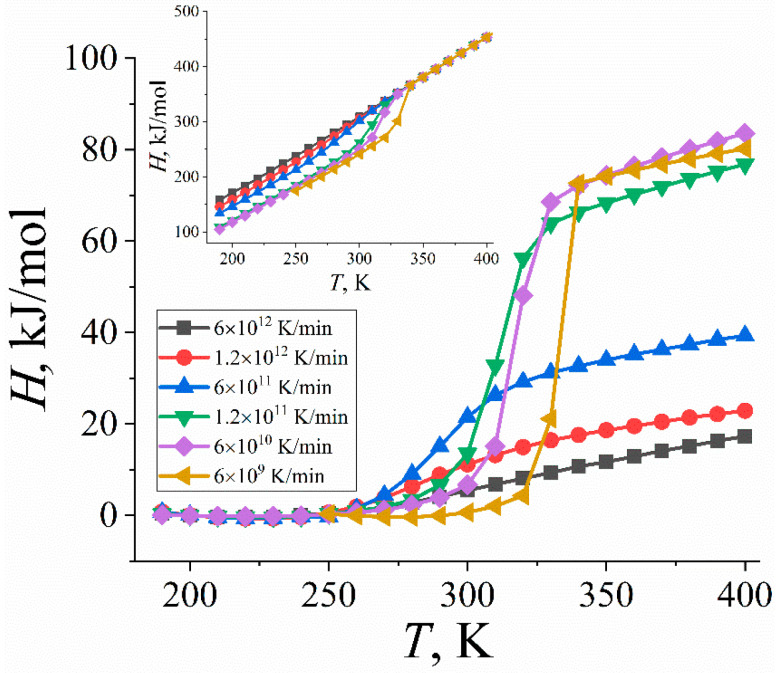
Enthalpy *H* of n-eicosane samples as a function of temperature *T* for different cooling rates. The enthalpy was reduced by subtracting the initial linear parts of the *H(T)*-curves in the low-temperature domain. The inset shows the original *H(T)*-curves.

The enthalpy change upon crystallization Δ*H_c_* is also of great importance for organic PCMs, as it is directly related to the accumulated heat released by the material. Experimentalists often focus on the changes in the enthalpy of paraffin samples during nonisothermal crystallization [5,7]. To evaluate the crystallization enthalpy Δ*H_c_* in computer simulations, the enthalpy of a system was calculated as *H* = *E_tot_* + *pV*, where *E_tot_* is the total (internal) energy of the system, *p* is pressure, and *V* is volume. The results for systems cooled down with different rates are shown in Figure 2. To determine the change in enthalpy upon phase transition, in DSC experiments, one often reduces the temperature dependence of the enthalpy to a zero level [24]. We did the same for the *H(T)*-curves by subtracting the initial linear parts of the curves in the low-temperature domain from the enthalpy (see Figure 2). The crystallization enthalpy Δ*H_c_* was then identified as the enthalpy after an abrupt change in the reduced *H(T)*-curves.

The crystallization enthalpies for paraffin samples cooled down with different rates are presented in Table 1. It is seen that faster cooling of an n-eicosane sample leads to a drop in the crystallization enthalpy in line with experimental data [7]. Interestingly, the crystallization enthalpies predicted by computer simulations employing slow cooling (6 × 10^9^, 6 × 10^10^, and 1.2 × 10^11^ K/min) are in excellent quantitative agreement with those measured in experiments (Table 1). It should be noted, however, that the observed agreement has to be taken with caution due to the tremendous difference in the cooling rate in simulations and experiments: a further decrease in the cooling rate in computer simulations can potentially lead to larger values of the crystallization enthalpy and, correspondently, to a larger deviation from experimental data. In turn, cooling with rates larger than 1.2 × 10^11^ K/min critically underestimates the crystallization enthalpy of n-eicosane, which implies that simulations with high cooling rates are not able to describe accurately the amount of heat accumulated by a paraffin sample.

Another important characteristic of organic PCMs is their thermal conductivity, as it controls the rate of charging and discharging of the PCM-based heat storage devices. It is known that the thermal conductivity coefficient *κ* of liquid paraffins (and n-eicosane in particular) is relatively low but increases considerably upon crystallization [25]. All these emphasize the importance of describing the thermal conductivity of PCMs through the cooling-rate simulations as accurate as possible.

**Figure 3:**
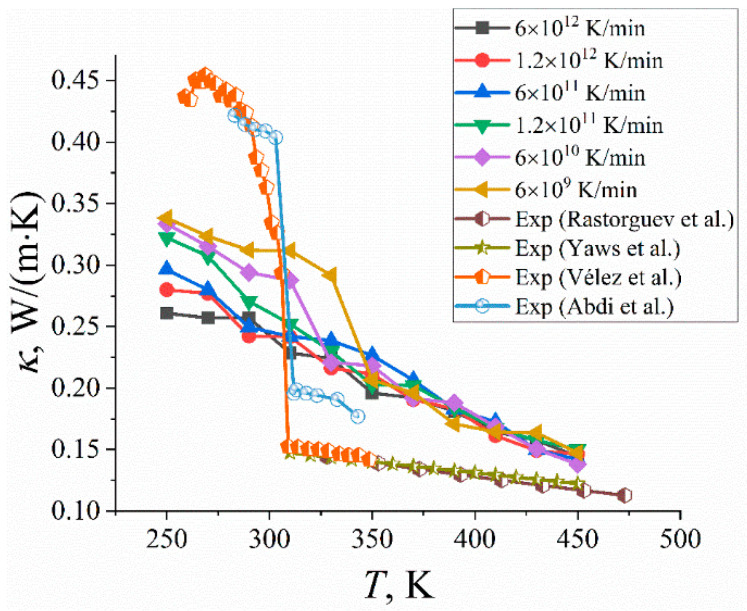
The thermal conductivity coefficient *κ* of n-eicosane samples as a function of temperature *T* for different cooling rates. For the sake of comparison, the experimental data are also shown [7,25,33,34].

Experimentally, it was shown that an increase in the cooling rate leads to a decrease in the κ value at a fixed temperature [26,27]. As for computer simulations, to the best of our knowledge, the impact of a cooling rate on thermal conductivity has not been studied by far. Most computational studies focused mainly on the thermal conductivity of crystalline paraffin samples at a fixed temperature [28] or on the temperature dependence of the thermal conductivity at a single cooling rate [29,30]. This is in part due to the performance limitations of the LAMMPS simulation package [31]: it is routinely used for evaluating thermal conductivity but not for cooling or heating a molecular system, as this is a very time-consuming and computationally demanding simulation. This can be overcome by means of a hybrid approach developed earlier by us [18]. It includes cooling a sample with the use of the highly efficient GROMACS package [32] and subsequent transformation of the simulated system to the LAMMPS representation for calculating the thermal conductivity coefficient at each temperature. Such an approach allowed us to explore for the first time the impact of cooling rate on the temperature dependence of the thermal conductivity of paraffins (see Figure 3).

First of all, it is seen that the *κ* value at a fixed temperature in the low-temperature domain systematically decreases with the increase in cooling rate, which is in qualitative agreement with the experiment [26,27]. As for the temperature dependence, the thermal conductivity coefficient *κ* shows steady growth with temperature for fast cooling of samples (cooling rates 6 × 10^11^, 1.2 × 10^12^, and 6 × 10^12^ K/min). In contrast, experiments report an abrupt jump in thermal conductivity upon crystallization [7,25,33,34]; see Figure 3. Therefore, the use of high cooling rates in computer simulations leads to an inaccurate description of the temperature dependence of thermal conductivity. This is most likely because fast cooling leads to partly crystallized paraffin samples at best (see Section 2.3). In turn, relatively slow cooling of samples with the rates of 6 × 10^9^, 6 × 10^10^, and 1.2 × 10^11^ K/min is able to qualitatively reproduce a jump in the thermal conductivity, although this jump is not as abrupt as in experiments (see Figure 3). The position of this jump is shifted to lower temperatures when the cooling rate increases. It should also be noted that the observed increase in the thermal conductivity for crystalline paraffin samples can be linked to the corresponding jump in the mass density (see Figure 1a) in line with earlier computer simulations [35]. Thus, our computational findings highlight for the first time the importance of a proper choice of a cooling rate for the accurate description of the thermal conductivity properties of paraffins.

### 2.2. Translational and Orientational Mobility

Besides thermophysical characteristics, the cooling rate can also affect the dynamics of paraffin molecules. In this Section, we explore its impact on the translational and local orientational mobility of n-eicosane chains.

Translational mobility is normally characterized by a diffusion coefficient. This quantity is easily accessible in a liquid paraffin sample but not in a crystal where the chain mobility is largely frozen [16,17]. Therefore, instead of calculating the diffusion coefficient, we chose to consider the mean square displacement (MSD) of paraffin chains at a certain time interval, as it allows us to characterize the translational mobility over the entire temperature range (250–450 K). The time interval at which MSD is measured should be the same for all systems at hand. The fastest cooling with the rate of 6 × 10^12^ K/min implies that the system is simulated for 100 ps at each temperature step of 10 K. Therefore, the time interval should not exceed 100 ps for all systems with different cooling rates. For our purposes, we discarded the last 20 ps of the intervals because of the rather high statistical uncertainty of the MSD curves at the end of a 100 ps trajectory.

**Figure 4:**
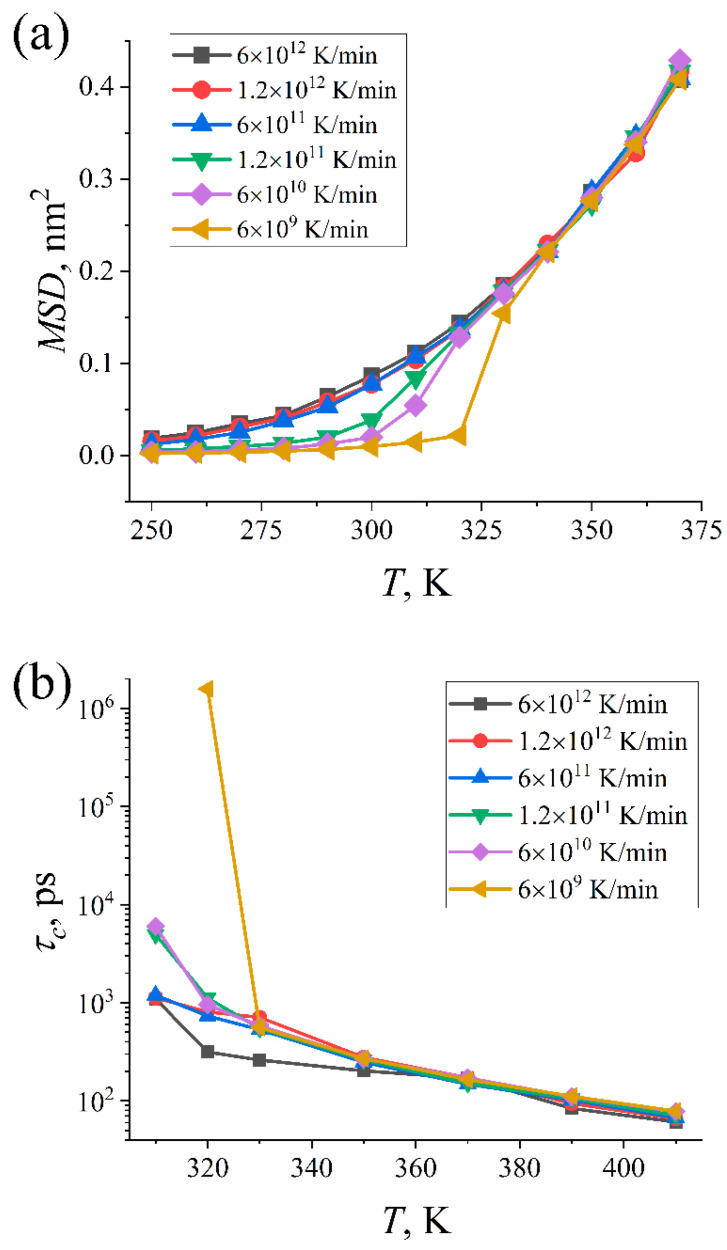
(**a**) Mean square displacements of paraffin molecules at a time interval of 80 ps as a function of temperature *T*. (**b**) The cumulative rotational relaxation time *τ_c_* of the C–C bond vector as a function of temperature *T*.

In Figure 4a, we present the mean square displacements at a time interval of 80 ps for paraffin samples cooled down with different rates (for clarity, the most interesting temperature interval from 250 to 375 K is shown). It is seen that the translational mobility of paraffin molecules is significantly slowed down at temperatures close to the crystallization temperature and almost fully suppressed in crystalline samples. The slower samples are cooled, the higher temperature at which this suppression occurs; this correlates with the impact of the cooling rate on the crystallization temperature, as discussed in the previous Section.

To characterize the local orientational mobility in paraffin samples, we computed the autocorrelation function *P*_1_*(t)* of the C–C bond vector:

(1) $$P_{1}(t)=\langle \vec{b}\left(t_{0}+t\right)\cdot \vec{b}\left(t_{0}\right)\rangle ,$$

where $\vec{b}$ is the unit vector along a C–C bond at times *t*_0_ and *t*_0_ + *t*, and the angle brackets 〈…〉 indicate averaging over both times *t*_0_ and all bond vectors in the system. The functional form of *P*_1_*(t)* can be fitted then by a sum of two Kohlrausch-Williams-Watts stretched exponents:

(2) $$f(t)=Ae^{-{\left(t/\tau_{1}\right)}^{\beta_{1}}}+\left(1-A\right)e^{-{\left(t/\tau_{2}\right)}^{\beta_{2}}},$$

where *A* ≤ 1, *τ*_1_ and *τ*_2_ are characteristic relaxation times, and *β*_1_ and *β*_2_ are parameters that account for non-exponential relaxation processes. Instead of *τ*_1_ and *τ*_2_, it is more convenient to consider a cumulative rotational relaxation time *τ_c_* defined as:

(3) τc=∫0∞P1(t)dt=Aτ1β1Γ(1β1)+(1−A)τ2β2Γ(1β2),

where Γ is the Gamma function.

In Figure 4b, we present the rotational relaxation time *τ_c_* for all considered cooling rates at relatively large temperatures. At *T* > 350 K, the temperature dependence of the rotational relaxation time *τ_c_* is linear, i.e., it follows the Arrhenius equation. The deviations from the linear dependence are observed at temperatures close to the crystallization temperature; similar behavior was also reported for the glass transition process of polymers [22]. These computational findings indicate the kinetic nature of the crystallization process at nanosecond time scales. When the temperature approaches the phase transition temperature, the rotational relaxation times increase considerably, which reflects a drop in the local orientational mobility of paraffin molecules upon crystallization. It is noteworthy that the temperature at which this drop occurs again depends on the cooling rate: smaller rates correspond to higher temperatures.

In addition, we explored the phonon transport in crystalline n-eicosane samples cooled down at different rates. To this end, we calculated the phonon vibrational density of state (VDOS) at *T* = 250 K (see Figure S2) [28]. We found that the intensities of VDOS for both carbon and hydrogen atoms increase when the cooling of samples becomes slower. In particular, decreasing the cooling rate from 6 × 10^12^ K/min to 6 × 10^9^ K/min results in additional pronounced peaks in the VDOS of carbon and hydrogen atoms (see Figure S2), indicating the appearance of new vibrational degrees of freedom, which can be used by acoustic phonons for propagation. These additional degrees of freedom could correlate with the formation of more ordered paraffin samples upon crystallization when slower cooling is applied. As we will see in the next Section, this is indeed the case.

### 2.3. Structural Properties of Crystalline Samples

**Figure 5:**
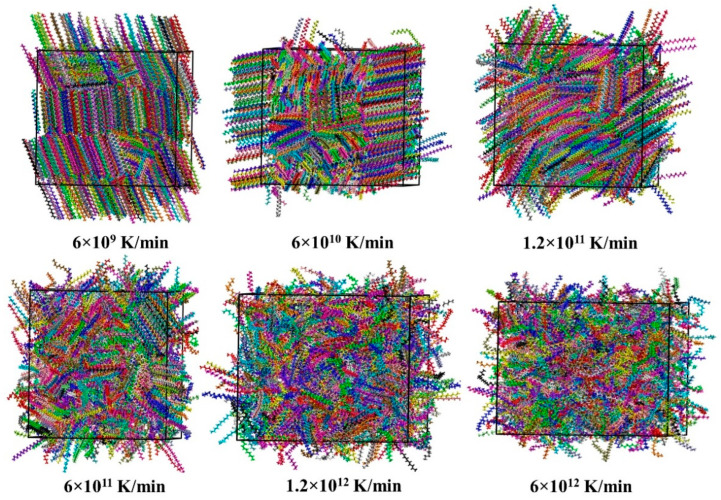
Snapshots of a simulation box with n-eicosane molecules at *T* = 250 K for different cooling rates.

Most computational findings discussed above suggest that the structure of crystalline paraffin samples at low temperatures should depend critically on the rate at which the samples are cooled down. The easiest way to see this is to inspect the corresponding snapshots (instantaneous configurations) of n-eicosane samples at *T* = 250 K (see Figure 5). Indeed, we observe progressively less ordered crystalline structures when speeding up the sample cooling. In the limiting case of the largest cooling rate of 6 × 10^12^ K/min, the crystallization of paraffin does not emerge.

A deeper insight into the structure of paraffin samples at low temperatures requires a special analysis aimed at evaluating which fraction of the samples are in the crystalline state. There is no unique way to estimate the crystallinity *χ* of a paraffin sample. Here we closely followed the approach proposed by Yamamoto et al. [36] and divided a simulation box into small cubic domains of size (2*σ*)^3^, where *σ* is the van-der-Waals radius of carbon atoms. In each cubic domain, we calculated the local orientational order parameter *P_2_(r)*:

(4) $$P_{2}\left(r\right)=\langle \frac{3}{2}\cos^{2}\theta -\frac{1}{2}\rangle ,$$

where *θ* is the angle between different vectors connecting the centers of neighboring C–C bonds within the same domain at position *r*, and the averaging is performed over all such vectors within the cubic domain. A domain is considered to be in the crystalline state if the corresponding orientational order parameter *P*_2_*(r)* exceeds 0.7 [36]. The crystallinity *χ* is then defined as a ratio of the number of crystalline domains to the total number of domains in the system. It is noteworthy that the results are found to remain almost the same if the threshold in the local orientational order parameter is increased to 0.85.

**Figure 6:**
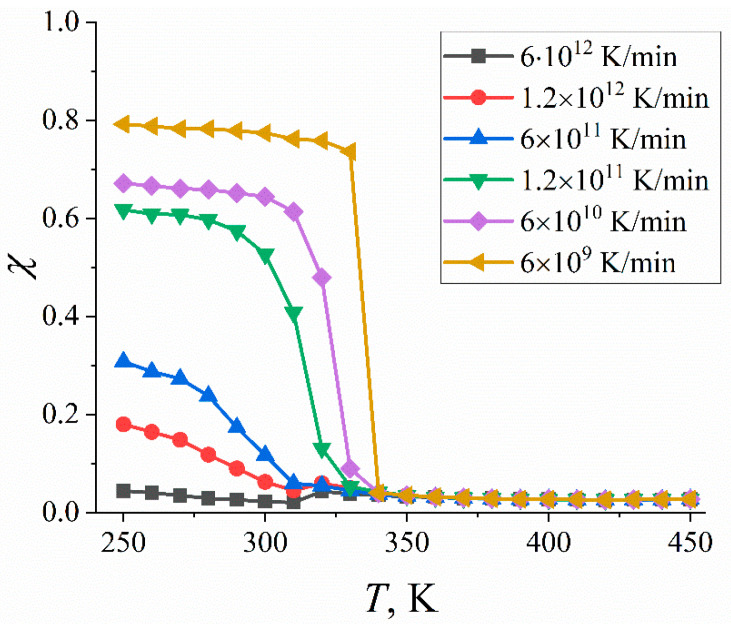
Crystallinity *χ* of n-eicosane samples as a function of temperature *T* for different cooling rates. The crystallinity is evaluated with the use of the approach of Yamamoto et al. [36].

In Figure 6, we show the temperature dependence of the crystallinity *χ* for different cooling rates. Apparently, at temperatures higher than the crystallization temperature, the crystallinity *χ* is equal to zero and insensitive to the cooling rate. At low temperatures, the crystallinity curves again can be divided into two groups corresponding to the slow (6 × 10^9^, 6 × 10^10^, and 1.2 × 10^11^ K/min) and fast (6 × 10^11^, 1.2 × 10^12^, and 6 × 10^12^ K/min) cooling; the former is characterized by an abrupt increase in the *χ* value upon crystallization. The crystallinity *χ* systematically decreases with the cooling rate. When the cooling rate is larger than 1.2 × 10^11^ K/min, the fraction of crystalline domains in the paraffin sample is less than one-third (see Figure 6). Thus, our findings show that fast cooling has to be avoided when studying the crystallization of paraffins.

It is also instructive to explore whether the temperature dependence of the crystallinity *χ* is sensitive to the method used for calculating *χ*. To this end, we repeated our calculations with the use of an alternative approach proposed by Triandafilidi et al. [37] (see Supplementary Materials for details). A comparison of Figure 6 and Figure S3 shows that both methods give similar results as far as the impact of the cooling rates on the crystallinity *χ* is concerned, although absolute values of *χ* may vary to some extent.

A comparison of the simulation values of crystallinity with experimental data is far from trivial. As discussed above, in computer simulations, the crystallinity is computed by evaluating a fraction of crystalline domains. In contrast, in experiments, the crystallinity is calculated from the enthalpy change Δ*H_c_* upon crystallization (see Table 1). If the crystallization enthalpy Δ*H*_ideal_ of an ideal crystal is known, then the experimental value of crystallinity is defined as *χ*_H_ = Δ*H*_c_/Δ*H*_ideal_ [38]. For an ideal n-eicosane crystal, the crystallization enthalpy is known to be 284.2 kJ/kg or 80.3 kJ/mol [23]. With this value at hand and with the use of experimental data available for the crystallization enthalpy of n-eicosane at different cooling rates [7], the crystallinity *χ*_H_ can easily be estimated (see Table 1). As for computer simulations, the crystallization enthalpy of an ideal n-eicosane crystal is not known. However, since the crystallization enthalpies found in simulations with slow cooling are in excellent agreement with experimental data (see Table 1), as a first approximation, one can use the experimental value of 80.3 kJ/mol for *ΔH*_ideal_. This makes it possible to compute the crystallinity *χ*_H_ for paraffin samples also in computer simulations, see Table 1. As one can anticipate from the crystallization enthalpy, the computed values of *χ*_H_ agree well with experimental data, provided that the cooling rate is smaller than 1.2 × 10^11^ K/min.

To conclude this Section, we focus on the structure of the n-eicosane crystalline phase. Experimentally, an n-eicosane sample undergoes various phase transformations upon cooling. At temperatures just below the melting point, n-eicosane enters the transient rotator phase [39]. Further cooling results in the formation of the triclinic crystalline structure of n-eicosane, which is characterized by both the positional and orientational order [40]. Therefore, it is crucial for computer simulations to reproduce the triclinic crystalline structure of n-eicosane samples in the low-temperature domain.

To characterize the crystal structure, we explored the relative orientations of n-eicosane molecules [16,41]. To this end, we calculated the probability distributions *P*(*θ*_1_, *θ*_2_) of relative orientation angles *θ*_1_ and *θ*_2_ for neighboring paraffin molecules (see Figure S4 for the definition of angles *θ*_1_ and *θ*_2)_.

**Figure 7:**
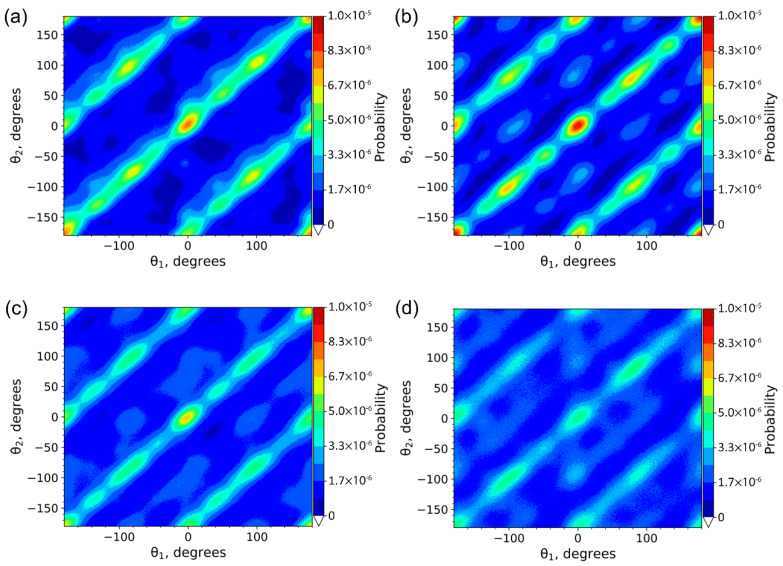
The probability distribution *P*(*θ*_1_,*θ*_2_) for n-eicosane crystalline samples cooled down with the rate of (**a**) 6 × 10^9^ K/min, (**b**) 6 × 10^10^ K/min, (**c**) 1.2 × 10^11^ K/min, and (**d**) 6 × 10^11^ K/min. Temperature is set to 250 K.

In Figure 7, we showed the probability distribution *P(θ*_1_*,θ*_2_*)* for crystalline n-eicosane samples cooled down with the four slowest rates from 6 × 10^9^ to 6 × 10^11^ K/min. A similar analysis for two other systems with very large cooling rates (1.2 × 10^12^ and 6 × 10^12^ K/min) is meaningless, as the crystalline phase is vanishing or completely suppressed. We found that for the systems with a so-called “slow cooling” (6 × 10^9^, 6 × 10^10^, and 1.2 × 10^11^ K/min), the *P(θ*_1_*,θ*_2_*)* maxima are localized on the main diagonal as well as on two sub-diagonals. As discussed in our previous paper [16], such a picture is typical for triclinic crystals, i.e., cooling-rate simulations are able to reproduce the ordering of n-eicosane chains correctly, provided that the cooling is sufficiently slow. What is more, the slower the cooling, the higher intensity of the maxima of the probability distributions *P(θ*_1_*,θ*_2_*)* presented in Figure 7; for the highest cooling rate of 6 × 10^11^ K/min, these maxima become considerably blurred. This implies that the triclinic crystalline structure of n-eicosane is less stable when the cooling rate increases. Note that there are additional maxima in the probability distribution *P(θ*_1_*,θ*_2_*)* outside the main diagonal and sub-diagonals (see Figure 7), implying that some n-eicosane molecules are still in the rotator phase. All the above-mentioned conclusions regarding the crystalline structure were drawn based on the analysis of a single initial configuration. We repeated our calculations for two more configurations (see Figures S5 and S6) and found very similar results.

As a final remark, we note that the threshold value of a cooling rate, which was reported in our study, should be taken with caution as it is related to n-eicosane (C_20_H_42_). The consideration of shorter n-alkanes most likely makes it possible to employ faster cooling in computer simulations as compared to n-eicosane because thermal relaxation occurs faster for shorter chains. In turn, slower cooling may be required for longer n-alkanes. Furthermore, the results reported in our study can also be relevant for the melting of paraffin samples, i.e., to the process that is opposite to the crystallization. In this case, one can expect the existence of the threshold value of a heating rate. This could be a subject of our future studies.

## 3. Methods and Materials

We performed molecular dynamics simulations of bulk samples of n-eicosane (C_20_H_42_), in which cooling is applied to a system at a constant rate (cooling-rate simulations). The all-atom force field GAFF [42] was chosen to describe paraffin chains. Recently, we showed that this force field provided a reasonable description of the thermophysical, structural, and dynamic properties of n-eicosane [16]. Furthermore, this force field was found to outperform 9 other atomistic force fields as far as thermal conductivity is concerned [18]. A paraffin sample consisted of 1000 n-eicosane chains, so the total number of atoms in a system amounted to 62,000.

According to experimental data, the melting temperature of n-eicosane is around 310 K [1]. Therefore, to observe the phase transition, most cooling-rate simulations were carried out over the interval from 450 to 250 K, although in some simulations, the cooling was additionally extended to 190 K. As the primary focus of our paper is to explore the impact of a cooling rate on the crystallization behavior of paraffine samples, all simulations were repeated with different cooling rates. In practice, the cooling of a sample was performed in a stepwise manner; the temperature step was always 10 K, but the simulation time at each temperature was systematically decreased from 100 ns to 100 ps. All in all, we considered 6 different cooling rates *γ_c_*: 6 × 10^9^, 6 × 10^10^, 1.2 × 10^11^, 6 × 10^11^, 1.2 × 10^12^, and 6 × 10^12^ K/min, spanning thereby four orders of magnitude. To improve the statistical reliability of the results, the simulations at each cooling rate were repeated several times with different initial configurations of a paraffin sample. Most cooling-rate simulations were independently carried out for 21 initial configurations, except for the system with the smallest cooling rate of 6 × 10^9^ K/min. For this system, the simulations are computationally demanding: a single cooling from 450 to 250 K requires a 2.1-µs-long run. Therefore, the simulations with the cooling rate of 6 × 10^9^ K/min were repeated for 12 configurations only. The initial configurations of a paraffin sample were extracted from a long trajectory of a system at *T* = 450 K [16]; special care was taken to ensure the statistical independence of the configurations.

The GROMACS package (v. 5) was used for cooling-rate molecular dynamics simulations [32]. The Nose-Hoover thermostat [43,44] and the Parrinello-Rahman barostat [45] were employed to control temperature and pressure in a system, respectively. The electrostatic interactions were handled by the particle mesh Ewald method [46]. The time step was 2 fs. The accumulated simulation time amounted to 30 µs.

To calculate the thermal conductivity, we used the LAMMPS simulation package [31,47]. As our cooling-rate simulations were carried out with the GROMACS suite, we first converted the paraffin samples from the GROMACS to LAMMPS representation using the approach developed in our earlier study [18]. After that, the thermal conductivity coefficient *κ* was evaluated from the heat flux autocorrelation function using the Green-Kubo relation [48]. This equilibrium molecular dynamics method was shown to be preferable over non-equilibrium counterparts for n-eicosane [18].

## 4. Conclusions

Here we report the first computational study that systematically explores how the cooling rate affects the accurate description of the crystallization of organic phase-change materials (paraffins) in atomic-scale molecular dynamics simulations. To achieve this, we systematically varied the cooling rate in computer simulations of n-eicosane from 6 × 10^9^ to 6 × 10^12^ K/min (we note that the cooling rate of 6 × 10^9^ K/min is close to the smallest rate accessible by most computational groups). We found the existence of the threshold value of 1.2 × 10^11^ K/min for cooling rates in simulations. When cooling is slower than the threshold, the simulations qualitatively reproduce an experimentally observed abrupt change in the temperature dependence of the density, enthalpy, and thermal conductivity of paraffins upon crystallization. Beyond this threshold, the paraffin’s properties observed in simulations deviate considerably from experimental data as the system is partly trapped in the supercooled liquid state. This can be witnessed through a systematic drop in the fraction of crystalline domains in a paraffin sample at low temperatures when the cooling rate increases. Furthermore, for fast cooling, we do not observe an abrupt change in the temperature dependence of the mass density, crystallization enthalpy, and thermal conductivity anymore. The crystallization temperature decreases systematically with the cooling rate in line with the experimental data available. Overall, our computational findings clearly show that a proper choice of a cooling rate is of tremendous importance in cooling-rate computer simulations of organic phase-change materials such as paraffins. Employing cooling rates that are too high could lead to a completely inaccurate description of the crystallization process as well as the thermophysical and structural properties of the system.

## Figures and Tables

**Table 1:** The crystallization temperature *T_c_*, the crystallization enthalpy Δ*H_c_*, and the crystallinity *χ_H_* = Δ*H_c_/*Δ*H_ideal_* (where Δ*H_ideal_* = 80.3 kJ/mol is the crystallization enthalpy for an ideal n-eicosane crystal [23]).

	*T_c_*, K	Δ*H*_c_*,* kJ/mol	χ_H_
*γ_c_* = 6 × 10^9^ K/min	332 ± 2	72.7 ± 1.0	0.91 ± 0.01
*γ_c_* = 6 × 10^10^ K/min	317 ± 2	68.6 ± 0.9	0.85 ± 0.01
*γ_c_* = 1.2 × 10^11^ K/min	312 ± 3	63.9 ± 1.0	0.80 ± 0.01
*γ_c_* = 6 × 10^11^ K/min	289 ± 3	29.2 ± 1.0	0.36 ± 0.01
*γ_c_* = 1.2 × 10^12^ K/min	280 ± 2	14.9 ± 1.0	0.19 ± 0.01
Experiment [1]	310	–	–
Experiment [7], *γ_c_* = 2.5 × 10^−2^ K/min	–	70.6	0.88
Experiment [7], *γ_c_* = 5 × 10^−2^ K/min	–	70.1	0.87
Experiment [7], *γ_c_* = 10^−1^ K/min	–	68.1	0.85

## Data Availability

The data presented in this study are available on request from the corresponding author.

## Supplementary Materials

The following supporting information can be downloaded at: https://www.mdpi.com/article/10.3390/ijms232314576/s1.

## Nomenclature

*ρ*: mass density (kg/m^3^); *C_p_*, molar specific heat capacity (kJ/(mol·K)); *T_c_*, crystallization temperature (K), Δ*H_c_*, crystallization enthalpy (kJ/mol); *χ_H_*, crystallinity (dimensionless); *γ_c_*, cooling rate (K/min); *H*, enthalpy (kJ/mol); *E_tot_*, total (internal) energy (kJ/mol); *p*, pressure (Pa); *V*, volume (nm^3^); *κ*, thermal conductivity coefficient (W/(m·K)); *τ_c_*, cumulative rotational relaxation time (ps); *P*(*θ*_1_, *θ*_2_) probability distribution (dimensionless).
