# Supplementary material for: Cooling-Rate Computer Simulations for the Description of Crystallization of Organic Phase-Change Materials
Source: Int J Mol Sci. 2022 Nov 23;23(23):14576. doi: 10.3390/ijms232314576 (PMC9737975; doi:10.3390/ijms232314576)
Supplement: Supplementary file 1 [file ijms-23-14576-s001.zip › ijms-2017841-supplementary.pdf]

Supplementary Materials

Cooling-Rate Computer Simulations for the Description of Crystallization of Organic Phase-Change Materials

Victor M. Nazarychev [1], Artyom D. Glova [1], Sergey V. Larin [1], Alexey V. Lyulin [2], Sergey V. Lyulin [1] and Andrey A. Gurtovenko [1,*]

1 Institute of Macromolecular Compounds, Russian Academy of Sciences, Bolshoi Prospect V.O. 31, 199004 St. Petersburg, Russia

2 Soft Matter and Biological Physics Group, Technische Universiteit Eindhoven, P.O. Box 513, 5600 MB Eindhoven, The Netherlands

* Correspondence: a.gurtovenko@gmail.com; Tel.: +7-(812)-3230216

1. Extended cooling of paraffin samples

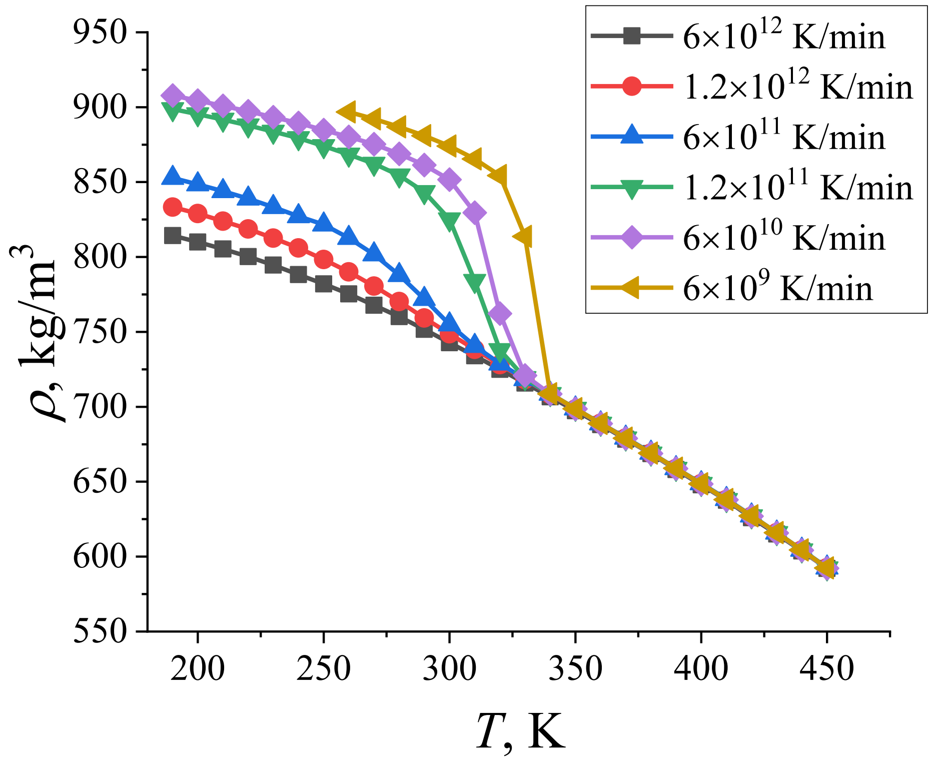

Figure S1. Mass density ρ of n-eicosane samples as a function of temperature T for different cooling rates. The cooling was extended to 190 K, except for the system with slowest cooling rate of 6×10^9 K/min.

2. Phonon vibration density of state

The phonon vibrational density of state (*VDOS*) was calculated as [1]:

$$VDOS(\omega) = \int_{-\infty}^{\infty} VACF(t) \cdot \exp(-2\pi i \omega t) dt \qquad \text{(S1)}$$

$$VACF(t) = \frac{\sum_{i=1}^{N} \langle v_i(t) \cdot v_i(0) \rangle}{\sum_{i=1}^{N} \langle v_i(0) \cdot v_i(0) \rangle} \qquad \text{(S2)}$$

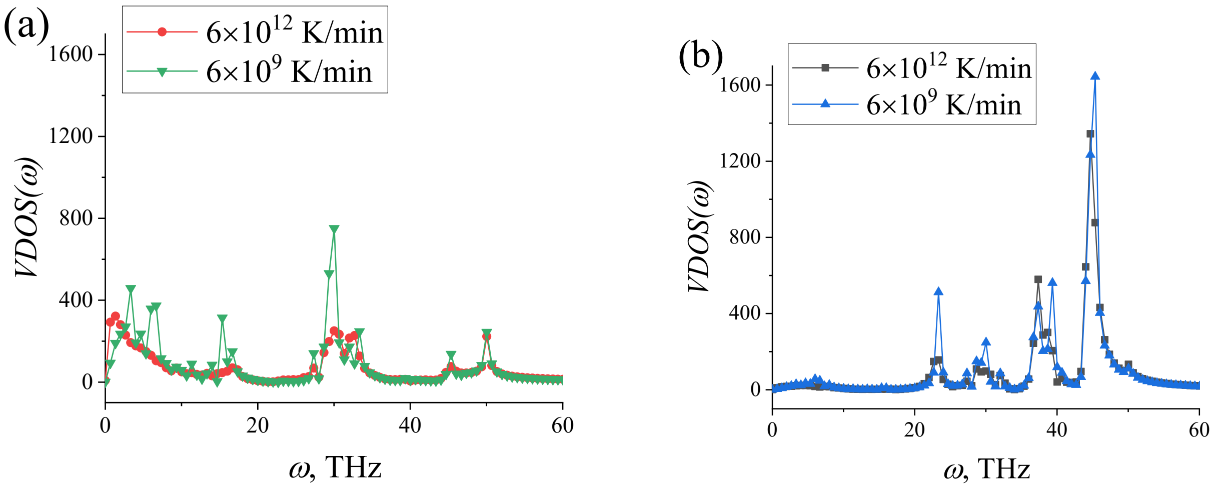

Figure S2. The phonon vibrational density of state (*VDOS*) of (a) carbon and (b) hydrogen atoms of n-eicosane samples cooled down with the rates of 6×10^9 and 6×10^{12} K/min. Temperature was set to 250 K.

3. Alternative approach for calculating crystallinity χ

An alternative approach for calculating the crystallinity χ was proposed by Triandafilidi et al. [2]. According to this approach, a simulation box is divided into cubic domains and for each domain a nematic tensor $Q^k_{\alpha\beta} = \left\langle b^k_{i,\alpha} b^k_{i,\beta} - \frac{1}{3}\delta_{\alpha\beta} \right\rangle$ is constructed (here **b** is the unit bond vector in cell k). Then the alignment director $\mathbf{s^k}$ (defined as the eigenvector corresponding to the largest eigenvalue) is calculated for each domain. The cubic domain is considered to be in the crystalline state if the condition $\frac{\mathbf{b^k_i} \cdot \mathbf{s^k}}{|\mathbf{b^k_i}||\mathbf{s^k}|} > 0.95$ is fulfilled. The crystallinity χ is defined as a ratio of the number of crystalline domains to the total number of domains.

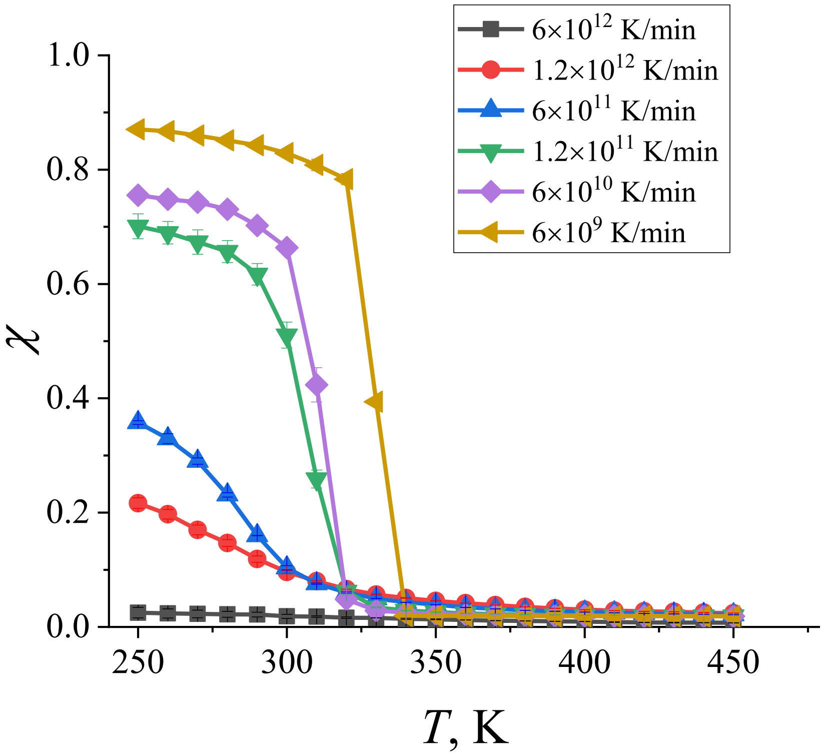

Figure S3. Crystallinity χ as a function of temperature T for n-eicosane samples for different cooling rates. The crystallinity is evaluated with the use of the approach of Triandafilidi et al. [2].

4. The probability distributions *$P(\theta_1,\theta_2)$*

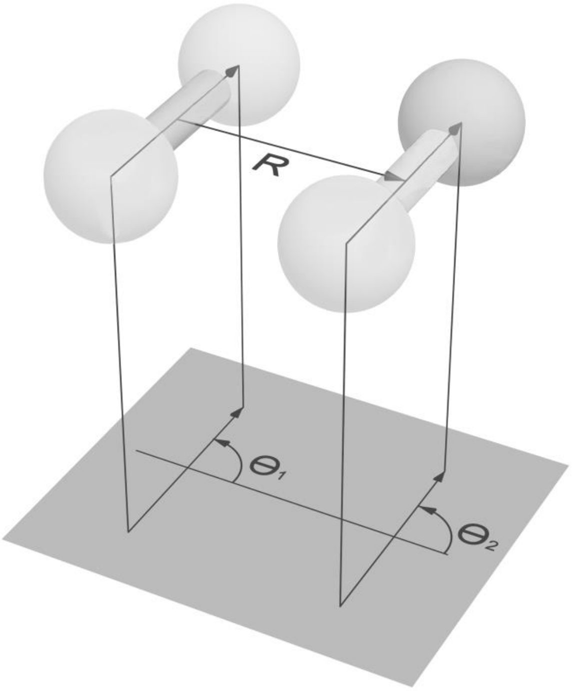

Figure S4. Definition of relative orientation angles θ_1 and θ_2 for neighboring monomers of two molecules in a crystalline n-eicosane sample [3,4].

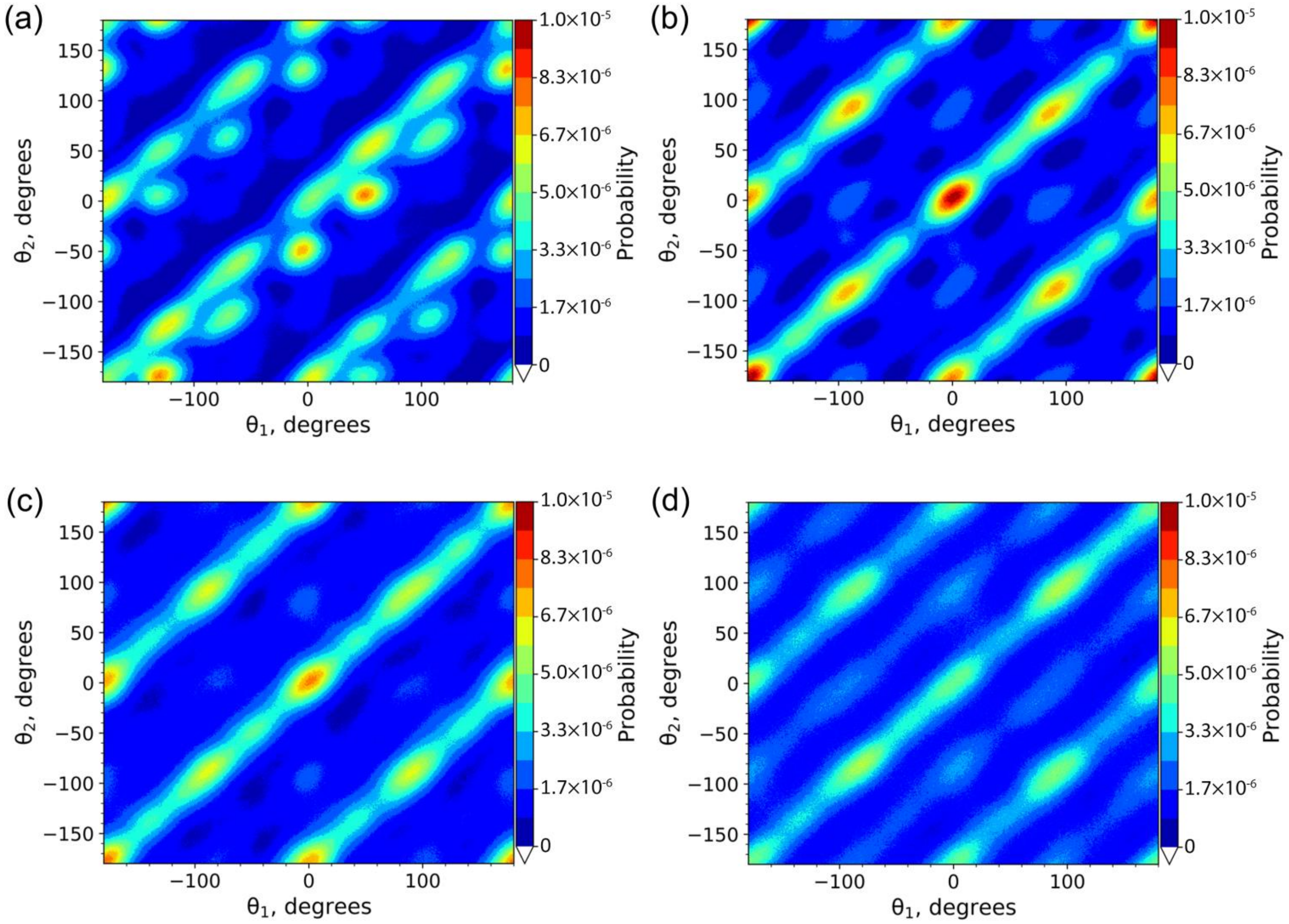

Figure S5. The probability distribution $P(\theta_1,\theta_2)$ for n-eicosane crystalline samples cooled down with the rate of (a) 6×10^9 K/min, (b) 6×10^{10} K/min, (c) 1.2×10^{11} K/min, and (d) 6×10^{11} K/min. Temperature is set to 250 K. Shown are the results for the second initial configuration of a system.

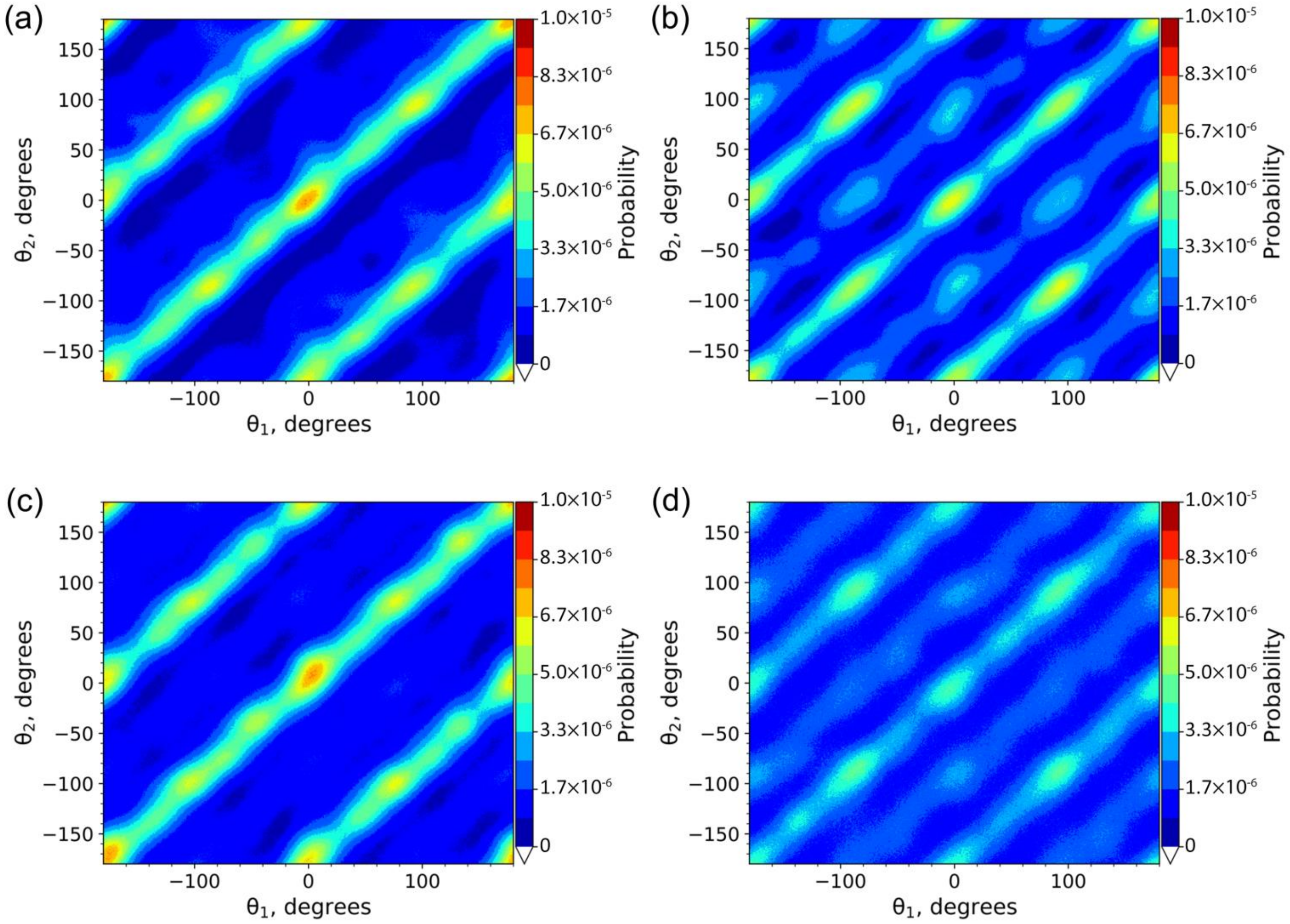

Figure S6. The probability distribution $P(\theta_1,\theta_2)$ for n-eicosane crystalline samples cooled down with the rate of (a) 6×10^9 K/min, (b) 6×10^{10} K/min, (c) 1.2×10^{11} K/min, and (d) 6×10^{11} K/min. Temperature is set to 250 K. Shown are the results for the third initial configuration of a system.

References

[1] S. Wu, H. Xie, W. Jiang, Q. Chen, Molecular dynamics study on the adsorption and thermal properties of paraffin in graphene, Int. J. Heat Mass Transf. 186 (2022) 122436. doi:10.1016/j.ijheatmasstransfer.2021.122436.

[2] V. Triandafilidi, J. Rottler, S.G. Hatzikiriakos, Molecular dynamics simulations of monodisperse/bidisperse polymer melt crystallization, J. Polym. Sci. B: Polym. Phys. 54 (2016) 2318–2326. doi:10.1002/polb.24142.

[3] A.D. Glova, I. V. Volgin, V.M. Nazarychev, S. V. Larin, S. V. Lyulin, A.A. Gurtovenko, Toward realistic computer modeling of paraffin-based composite materials: critical assessment of atomic-scale models of paraffins, RSC Adv. 9 (2019) 38834–38847. doi:10.1039/C9RA07325F.

[4] N. Wentzel, S.T. Milner, Simulation of multiple ordered phases in C23 n-alkane, J. Chem. Phys. 134 (2011) 224504. doi:10.1063/1.3589417.
